# mTORC1 regulates phagosome digestion of symbiotic bacteria for intracellular nutritional symbiosis in a deep-sea mussel

**DOI:** 10.1126/sciadv.adg8364

**Published:** 2023-08-23

**Authors:** Akihiro Tame, Tadashi Maruyama, Tetsuro Ikuta, Yoshihito Chikaraishi, Nanako O. Ogawa, Masashi Tsuchiya, Kiyotaka Takishita, Miwako Tsuda, Miho Hirai, Yoshihiro Takaki, Naohiko Ohkouchi, Katsunori Fujikura, Takao Yoshida

**Affiliations:** ^1^ Research Institute for Global Change, Japan Agency for Marine-Earth Science and Technology, 2-15 Natsushima-cho, Yokosuka, Kanagawa 237-0061, Japan.; ^2^ School of Marine Biosciences, University of Kitasato, Minami-ku, Sagamihara, Kanagawa 252-0373, Japan.; ^3^ Faculty of Medical Sciences, Life Science Research Laboratory, University of Fukui, 23-3 Matsuoka Shimoaizuki, Eiheiji-cho, Yoshida-gun, Fukui 910-1193, Japan.; ^4^ Institute of Low Temperature Science, Hokkaido University, Kita-19, Nishi-8, Kita-ku, Sapporo 060-0819, Japan.; ^5^ Research Institute for Marine Resources Utilization, Japan Agency for Marine-Earth Science and Technology, 2-15 Natsushima-cho, Yokosuka, Kanagawa 237-0061, Japan.; ^6^ Department of Environmental Science, Fukuoka Women's University, Kasumigaoka 1-1-1, Higashi-ku, Fukuoka 813-8529, Japan.; ^7^ Institute for Extra-cutting-edge Science and Technology Avant-grade Research, Japan Agency for Marine-Earth Science and Technology, 2-15 Natsushima-cho, Yokosuka, Kanagawa 237-0061, Japan.

## Abstract

Phagocytosis is one of the methods used to acquire symbiotic bacteria to establish intracellular symbiosis. A deep-sea mussel, *Bathymodiolus japonicus*, acquires its symbiont from the environment by phagocytosis of gill epithelial cells and receives nutrients from them. However, the manner by which mussels retain the symbiont without phagosome digestion remains unknown. Here, we show that controlling the mechanistic target of rapamycin complex 1 (mTORC1) in mussels leads to retaining symbionts in gill cells. The symbiont is essential for the host mussel nutrition; however, depleting the symbiont’s energy source triggers the phagosome digestion of symbionts. Meanwhile, the inhibition of mTORC1 by rapamycin prevented the digestion of the resident symbionts and of the engulfed exogenous dead symbionts in gill cells. This indicates that mTORC1 promotes phagosome digestion of symbionts under reduced nutrient supply from the symbiont. The regulation mechanism of phagosome digestion by mTORC1 through nutrient signaling with symbionts is key for maintaining animal-microbe intracellular nutritional symbiosis.

## Teaser

Endosymbiosis in deep-sea mussels is sustained by inhibiting phagosome digestion via nutrient signaling with symbionts.

## INTRODUCTION

Establishing intracellular symbiosis with bacteria ensures the availability of a wide range of nutrients to the hosts. It leads to prosperous and diverse biotas (*1*), even in nutrient-limited environments like the deep sea (*1*, *2*). Numerous host animals, especially those that live in marine habitats, acquire symbiotic microbes from the environment through horizontal transmission in each generation (*3*–*6*). Phagocytosis in eukaryotic cells, a process ranging from engulfment to intracellular digestion of the microbes (*7*, *8*), has been thought to contribute to horizontal transmission (*1*–*4*). In innate immunity, host immune cells, such as macrophages, phagocytose exogenous microbes into phagosomes and digest them using lysosomal hydrolases in phagolysosomes developed through a phagosome maturation process (*7*). Some horizontally transmitted symbiotic microbes, having the host-targeting type III, IV, and VI secretion systems, avoid digestion by inhibiting phagosome maturation using inhibitory factors excreted by these secretion systems and survive within the host cells (*3*, *9*–*11*). In these symbioses, the secretion systems in symbiotic microbes are thought to maintain symbiosis by blocking phagosome digestion in the hosts. One notable exception is the intracellular symbiosis in deep-sea *Bathymodiolus*/*Gigantidas* mussels, which have a symbiotic relationship with chemosynthetic bacteria. Among the species belonging to this genus, *Bathymodiolus japonicus* harbors host species-specific methane-oxidizing symbiotic bacteria (from now on referred to as symbionts) in the vacuoles (symbiosomes) of gill epithelial cells (bacteriocytes) (*2*, *12*). The mussel gill cells have phagocytic ability (*13*); however, genes involved in host-targeting secretion systems have not been found in the genomes of symbionts of *Bathymodiolus* mussels, including *B. japonicus* (*14*–*17*). Nevertheless, the bacteriocytes phagocytose and digest exogenous bacteria, including dead symbionts, while the symbionts are retained in symbiosomes (*13*). This suggests that the bacteriocyte has a mechanism to discriminate the resident living symbionts from those of other exogenous bacteria, including the dead symbiont. The latter bacteria are selectively digested in their phagosomes. This selection mechanism must be fundamental to sustaining intracellular symbiosis; however, it is an enigma that needs to be addressed.

*Bathymodiolus* mussels are known to rely largely on symbionts for nutrition (*5*, *15*–*18*). When mussels were reared in an aquarium without a symbiont’s energy and carbon source, such as methane, they lost the symbionts in their bacteriocytes leading to a decline in their overall health status (*19*, *20*). Therefore, some nutrient supply from its symbiont is likely essential for maintaining symbiosis in mussels. The acquisition of nutrients from symbionts in *Bathymodiolus* mussels is still a matter of debate, and two pathways, the digestion of symbionts (farming) and receipt of secreted metabolites from symbionts (milking), have been proposed (*15*, *16*, *18*). Recently, we found that most of the symbionts in freshly collected *B. japonicus* were maintained in the symbiosomes without digestion, suggesting that the host mussels may directly receive metabolites from symbionts in bacteriocytes (*13*).

The mechanistic target of rapamycin complex 1 (mTORC1), which localizes on the lysosomal surface, functions as a central regulator of cell metabolism through sensing amino acids and sterols inside lysosomes (*21*). mTORC1 plays crucial roles in lysosomal function by regulating the biogenesis, distribution, and activity of lysosomes, as well as in the coordination of both anabolisms, such as protein and cholesterol biosynthesis, and catabolism like protein degradation (*21*–*24*). Transcripts of genes in the mTOR pathway have also been identified in the gills of *Bathymodiolus* mussels, regardless of the mussel species or habitat (*17*). From these, we hypothesized that mTORC1, which is known to regulate lysosomal digestion in cellular metabolism, acts as a mediator for sensing nutrients from symbionts and regulating phagosome digestion against symbionts in the bacteriocytes of host mussels.

The present study aimed to clarify the potential regulatory role of mTORC1 in nutrient sensing and phagocytosis to sustain intracellular nutritional symbiosis in the mussel *B. japonicus*. The sequence analysis of dual transcriptomes of host mussels and symbionts and traditional bulk nitrogen and carbon isotope analyses were performed to investigate the nutritional relationships between the host mussel and symbiont. In addition, using the nitrogen isotopic composition of amino acids to estimate food sources and trophic position (TP), we examined changes in the trophic level of the host before and after the disappearance of symbiont during mussel rearing in an aquarium lacking energy and carbon sources for the symbiont. We also examined the localization of mTORC1 and phagocytic digestion-related factors in mussel gill cells using immunohistochemistry with antibodies against these factors in *B. japonicus*. To investigate the involvement of mTORC1 in phagosome digestion, we examined the effect of its inhibitor, rapamycin, on the disappearance of symbionts during rearing. We also examined the changes in the digestion state of symbionts using immunohistochemistry and enzyme histochemistry. Last, we investigated whether mTORC1 is involved in the selective regulation of phagosome digestion between resident and exogenous dead symbionts in gill cells.

## RESULTS AND DISCUSSION

### Nutritional relationship between *B. japonicus* and its symbiont

**Fig. 1.**
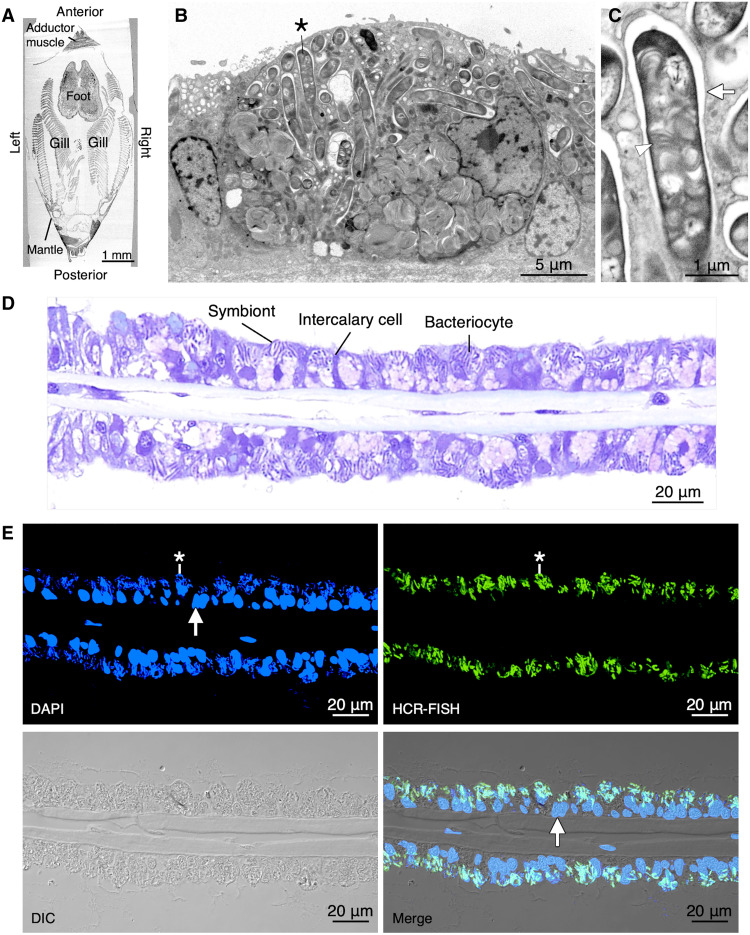
Localization of the methane-oxidizing symbionts in the bacteriocytes of *B. japonicus*. (**A**) SEM micrograph of a cross section (ventral view) through the entire small mussel (shell length of about 7 mm) shows the mussel body structure. (**B**) SEM micrograph in the gill site shows a bacteriocyte harboring symbionts. An asterisk represents a typical rod-shaped symbiont. (**C**) A higher-magnification SEM micrograph shows that the symbiont had a stacking membrane structure (arrowhead) in the symbiosome (arrow). The SEM micrographs are the reversed backscattered electron images. (**D**) Bright-field micrograph of a toluidine blue–stained gill filament shows the distribution of bacteriocyte-harbored symbionts and other gill cells. (**E**) Fluorescence, differential interference contrast (DIC), and merged (Merge) micrographs of a gill section show that the symbionts stained with DAPI (blue) were also detected by HCR-FISH with the specific probes (green). Arrow indicates a nucleus of a host gill cell. Asterisks represent a symbiont.

*B. japonicus* harbors specific methane-oxidizing bacteria, which have a stacking membrane in the cytoplasm, within the symbiosomes of bacteriocytes in the gills (Fig. 1, A to D). The rod-shaped symbionts that were detected using hybridization chain reaction fluorescence in situ hybridization (HCR-FISH) with specific methane-oxidizing bacteria probes were also stained with 4′,6-diamidino-2-phenylindole (DAPI) (Fig. 1E). The rod-shaped symbionts stained with DAPI were defined as methane-oxidizing symbiotic bacteria. Transcriptome analysis has previously been conducted on other *Bathymodiolus* mussels to determine the nutrient interactions between the host and symbionts (*15*–*17*). It has been found that several metabolic systems, including amino acid and sterol synthesis, are complemented between the host and symbionts, and the factors, such as biosynthetic enzymes, associated with them have been found. We investigated this symbiotic relationship using a dual transcriptome analysis of *B. japonicus* and its symbiont. The statistics for the study are summarized in table S1. Dual RNA sequencing (RNA-seq) of the mussel gills produced 156,890,244 reads after the cleanup processes. The reads were assembled to yield transcriptomes of the mussel and symbiont. Overall, 241,317 nonredundant transcripts were identified, and 49,998 protein-coding transcripts were found. Among these protein-coding transcripts, 28,880 were assigned to the phylum Mollusca as the host transcriptome. Moreover, 31,225 transcripts were also homologous to the genes in *Gigantidas platifrons* genomes. Benchmarking Universal Single-Copy Orthologs (BUSCO) analysis showed 96.0 and 85.6% completenesses for the metazoa odb10 and mollusca odb10 databases, respectively. In contrast, the order Methylococcales, as the symbiont, contained 1353 transcripts with a total size of 3,515,217 base pairs (bp), which showed 81.0% completeness for the gammaproteobacteria odb10 database. The percentage of reads mapped to the Mollusca and Methylococcales transcripts was 82.7 and 12.3%, respectively (fig. S1A). The distribution of transcript expression levels of Mollusca and Methylococcales showed that the transcripts per million in each transcriptome were of similar order (approximately 1 × 10^1^) (fig. S1B).

The dual transcriptome data showed that, as with other mussels (*16*), the symbiont had genes for synthesizing sterol intermediates from methane, while the gills of mussels had genes associated with the downstream steps of the steroid biosynthesis pathway (table S2 and fig. S1C). In addition, the mussel gills and symbionts appeared to complement each other in their respective amino acid biosyntheses (table S2 and fig. S1D). The mussel gills had major genes involved in transporting sterols and amino acids (table S3). The symbionts have genes for some compound transporters, such as ATP-binding cassette (ABC) transporters, and for bacterial secretion systems, including type II (table S3), known to export various proteins in an extracellular manner (*25*). The genes of factors related to the inhibition of phagosome digestion and other secretion systems, such as type III, IV, and VI secretion systems, were not found in the transcripts of symbionts in *B. japonicus*. We also surveyed the genome data of the symbiont of *B. japonicus* (accession no. BLYC01000001 to BLYC01000109). These genes were not found in the symbiont’s genome or in other *Bathymodiolus/Gigantidas* mussels (*14*). The genes for steroid and amino acid biosynthesis were transcribed in the gills and symbionts, along with the genes for ribosomal protein metabolism and central metabolism, including glycolysis, tricarboxylic acid cycle, and pentose-phosphate cycle (fig. S1E). Our data suggested that symbionts complement the steroid biosynthesis and amino acid biosynthesis of the host mussel, respectively. Further metabolic and biochemical analyses are necessary to verify the transports of the nutrients between the symbiont and host in more detail.

### Changing the nutritional relationship through depleting energy and carbon source of symbiont

**Fig. 2.**
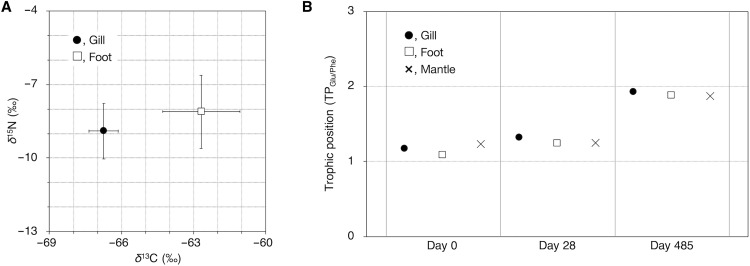
Stable isotopic analysis of *B. japonicus* during mussel rearing. (**A**) Estimated bulk carbon (δ^13^C) and nitrogen (δ^15^N) signatures at rearing day 0. δ^13^C values showed statistically significant (*P* < 0.05) differences between the gill and foot, whereas δ^15^N values showed no significant differences (*P* > 0.05). Bars indicate means ± SD from three independent experiments using three mussels. (**B**) Estimated TP (TP_Glu/Phe_) value during mussel rearing. The TP_Glu/Phe_ value was estimated from the nitrogen isotopic composition analyzing individual amino acids (δ^15^N) using tissues of gill, foot, and mantle from mussels after rearing for 0, 28, and 485 days (*n* = 1).

While the methane-oxidizing symbiont oxidizes and metabolizes methane to produce energy and nutrients, the *Bathymodiolus* mussels have been thought to obtain nutrients by intracellularly digesting symbionts as feed or by receiving secreted compounds from the symbiont (*15*, *16*, *18*). To examine the trophic relationship and resources, we performed stable carbon and nitrogen isotopic analyses of bulk samples from fresh mussels immediately after collection. In *B. japonicus*, the isotopic differences between gills, including symbionts and foot as an asymbiotic tissue, were 4.1 ± 1.0‰ (−66.7 ± 0.6‰ for gill and −62.7 ± 1.6‰ for foot) for carbon and 0.8 ± 0.4‰ (−8.9 ± 1.1‰ for gill and −8.1 ± 1.5‰ for foot) for nitrogen (Fig. 2A). The low δ^13^C range in both gills and foot strongly suggested that their carbon source was of biogenic methane origin (*26*). In contrast, no notable ^15^N enrichment in the gills relative to the foot indicated no substantial trophic relationship between them (*27*).

A previous study has reported that the adult *Bathymodiolus* mussels lost the symbionts during the 30-day rearing without any energy sources for the symbionts (*20*). In the present study, similar to the previous report, when the mussels were reared under methane-depleted conditions, the symbionts decreased on day 28 and completely disappeared on day 485 (fig. S2A). By transmission electron microscopy (TEM), while the bacteriocytes of mussels harbor many intact symbionts before the rearing (fig. S2B), the number of symbionts apparently decreased in those of mussels reared for 28 days (fig. S2C). In addition, on day 28, the rod-shaped symbionts were highly vacuolated (fig. S2, C to E). This may indicate that the symbionts were digested in the symbiosomes during the rearing methane-depleted condition. To examine the nutritional status of mussels during rearing in the absence of methane, we conducted the nitrogen isotopic analysis of individual amino acids and estimated the TP based on the δ^15^N values of glutamic acid and phenylalanine (*28*). The increase in trophic level has little effect on the carbon isotope ratio. However, the nitrogen isotope ratio changes greatly, and therefore, the nitrogen isotopic analysis helps investigate the TP and determine whether it is autotroph or heterotroph (*28*). Heterotrophically grown bacteria elevate one trophic unit relative to their diet, as in a predator-prey grazing relationship, whereas autotrophically grown microbes exhibit TP 1, like autotrophs (*29*, *30*). For example, in the case of photosymbiotic giant clams nutritionally dependent on their intracellular symbiotic algae, TP for the alga is estimated to be 0.9 but that for the host clam adductor muscle as a symbiont-free tissue is 2.0, suggesting that most nitrogen is obtained by digesting symbiotic algae (*31*). In the present study, the TPs of the examined tissues in the mussel, including the symbiont, before the onset of rearing were 1.1 to 1.3 (Fig. 2B), which was close to the values of autotrophs (*28*). After rearing on day 28, the TPs were 1.2 to 1.3; however, after rearing on day 485, the values increased to ~1.9 (Fig. 2B). Our previous observations showed that some symbionts are enzymatically digested in bacteriocytes (*13*). This increase in TPs on day 485 implied that the mussels obtained nitrogen by intracellularly digesting symbionts in bacteriocytes. These results indicate that the nutritional relationship in the mussel-symbiont shifted the nutritional style from secretion by symbionts (milking) to the digestion of symbionts (farming) during rearing. It was assumed that the TP values did not change significantly until day 28 because the mussel bacteriocytes probably initiated the digestion of the symbionts at this time. The host nitrogen metabolism has likely not yet sufficiently changed from using the secretions to metabolizing the digested products. Taking into consideration that mussels use nutrients, such as methane-derived carbon compounds, including steroids and amino acids and/or their intermediates, supplied by the symbiont before rearing (Fig. 2A), due to methane depletion, the decrease in nutrient supply from the symbiont may switch on the digestion of the symbiont to supplement the host’s nutrition. Hence, it is suggested that the nutrient supply from the symbiont to the mussel is involved in suppressing the intracellular digestion of symbionts.

### Promoting the phagosome digestion of symbionts by mTORC1 under methane-depleted conditions

**Fig. 3.**
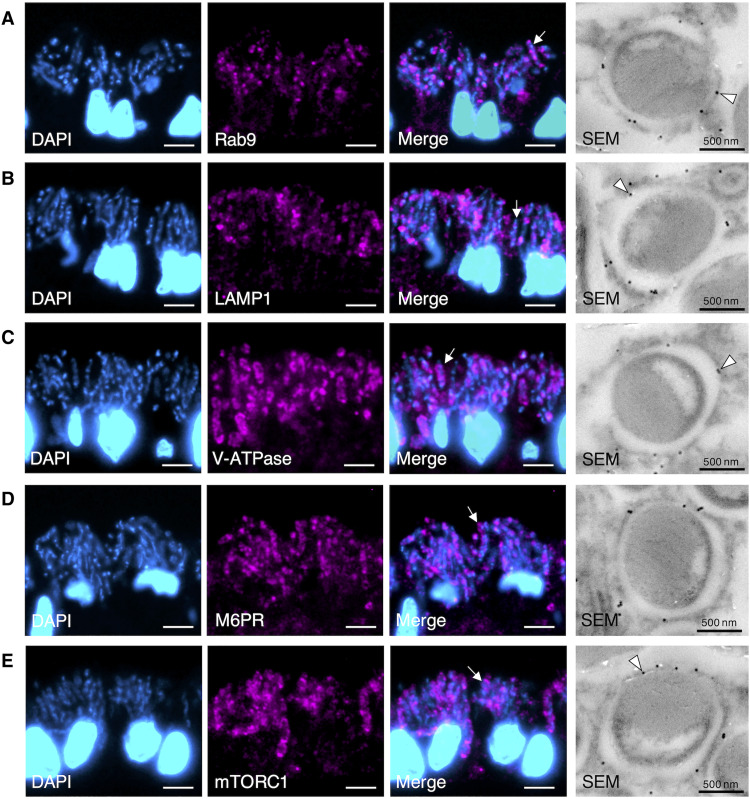
Immunohistochemistry of the symbiosomes harboring the symbionts. Immunofluorescence micrographs show the symbionts stained with DAPI (blue) and symbiosomes stained with antibodies (magenta) against Rab9 (**A**), LAMP1 (**B**), V-ATPase (**C**), M6PR (**D**), and mTORC1 (**E**) in bacteriocytes. SEM micrographs (right) show immunogold localization (black dots, arrowheads) of the antibodies on the symbiosome in mussels at rearing day 0. Arrows indicate antibody-immunostained symbiosome harboring symbiont. Scale bars, 5 μm.

The mTORC1 on the lysosomal surface is a center for regulating various cellular metabolisms through lysosomes by sensing nutrients inside the lysosome, such as cholesterol and amino acids (*22*, *24*, *32*, *33*). Until now, in the gill cells of *B. japonicus*, only a small fraction of the resident symbionts is enzymatically digested by esterase during a 24-hour incubation with other bacteria in mussels (*13*). In the present transcriptome analysis, we found transcripts of major genes associated with mTORC1 and phagosome digestion pathways in the gills (table S4). These genes involved in these pathways were present and evolutionarily conserved in the genomes of bivalves, including *G. platifrons* (table S5). To clarify whether the symbiosomes harboring symbionts have phagosome digestion-related factors and mTORC1, we prepared antibodies against the peptides corresponding to Ras-related protein Rab-9 (Rab9), lysosomal-associated membrane protein 1 (LAMP1), vacuole-type H+ adenosine triphosphatase (V-ATPase), mannose-6-phosphate receptor (M6PR), and mTORC1, based on the amino acid sequences in the gill of *B. japonicus*. Immunohistochemical analysis revealed that Rab9, LAMP1, V-ATPase, M6PR, and mTORC1 were detected in symbiosomes harboring symbionts in the bacteriocytes of fresh host mussels (Fig. 3). The phagosome digestion process is initiated by engulfing and terminated by excluding the residual material according to the following process (*7*, *34*, *35*): (i) phagosomes containing bacteria mature to phagolysosomes by binding some phagosome regulator proteins, such as Rab9 and LAMP1; (ii) M6PR delivers newly synthesized lysosomal hydrolases from the trans-Golgi network to the phagolysosome; and (iii) the transported hydrolases are activated in phagolysosomes acidified by proton translocation via V-ATPase and then digest the bacteria. It has been reported that mTORC1 is activated on the lysosomal surface through cellular amino acid stimulation, whereas inactive mTORC1 is located in the cytoplasm (*24*). The present results showed that the symbiosomes harboring resident symbionts had mTORC1, suggesting that they were characterized as phagolysosomes (Fig. 3E).

**Fig. 4.**
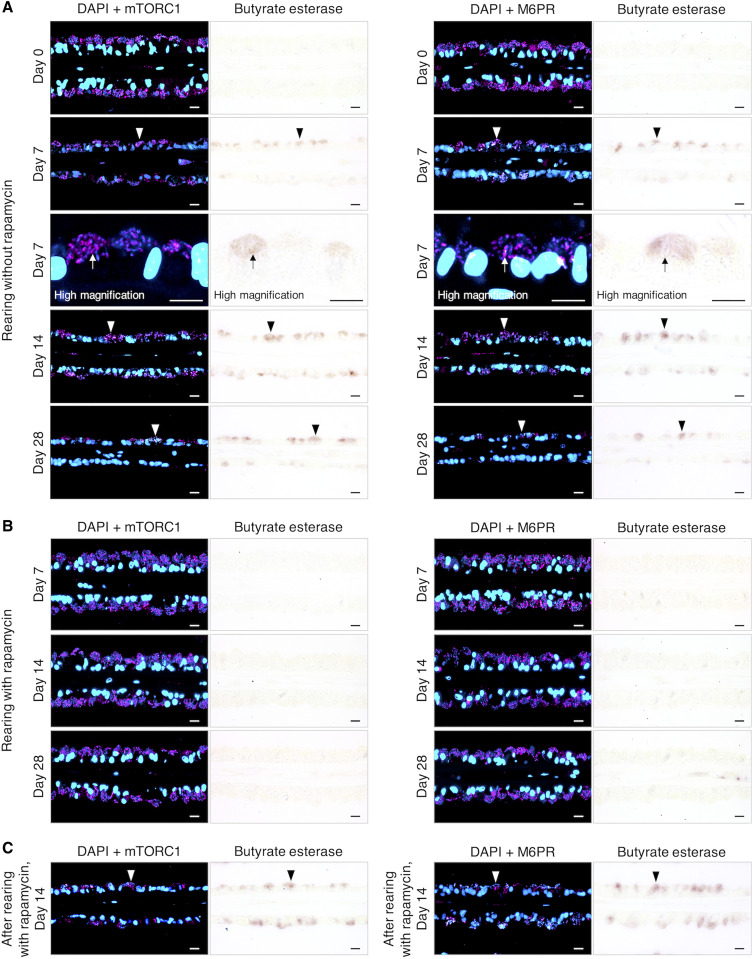
Immunohistochemistry and butyrate esterase histochemistry of gill cells from the mussels reared with or without rapamycin. (**A**) Merged fluorescence micrographs of DAPI (blue)–stained symbiont cells in symbiosomes, which were concomitantly stained with antibodies (magenta) against mTORC1 (left) or M6PR (right) in gill sections from the mussel reared for 0, 7, 14, and 28 days in the absence of rapamycin under methane-depleted condition. Bright-field micrographs show histochemical staining for butyrate esterase activity (brownish red) in the symbiosomes of bacteriocytes, of which tissue sections were respectively the same for the fluorescence micrographs. High-magnification micrographs on day 7 show colocalization of symbiont (DAPI) and butyrate esterase activity in some symbiosomes bound to mTORC1 or M6PR (arrows). (**B**) Merged fluorescence micrographs of symbiosomes with DAPI-stained symbionts and those stained with antibodies (magenta) against mTORC1 (left) or M6PR (right) in gill sections from the mussel reared with 2.8 μM rapamycin under methane-depleted condition for 7, 14, and 28 days. Bright-field micrographs show no butyrate esterase activity in bacteriocytes in the same sections used for fluorescence micrographs. (**C**) Mussels were reared in the methane-depleted aquarium with rapamycin for 14 days and then without rapamycin for 14 days. Merged fluorescence micrographs show the sectioned gill epithelia stained with DAPI and with antibodies (magenta) against mTORC1 (left) or M6PR (right). Bright-field micrographs show positive butyrate esterase activity in symbiosomes of bacteriocytes on the same tissue sections as those used for respective fluorescence micrographs. Arrows indicate a symbiosome showing colocalizations of mTORC1 (left) or M6PR (right) and of butyrate esterase activity. Arrowheads indicate the bacteriocytes exhibiting butyrate esterase activity in symbiosomes harboring DAPI-stained symbionts bound to mTORC1 (left) or M6PR (right). Scale bars, 10 μm.

To investigate whether the symbionts were digested in symbiosomes through phagosome digestion under methane-depleted conditions, the mussels were reared in a methane-depleted aquarium for 7, 14, and 28 days, and esterase activity was analyzed along with the localization of M6PR and mTORC1 in the mussel gill cells. Before rearing, no butyrate esterase activity was detected in the bacteriocytes, while mTORC1 and M6PR were abundantly detected in the symbiosomes (day 0 in Fig. 4A). After day 7, butyrate esterase activity was detected in the symbiosomes (arrows at day 7, high magnification in Fig. 4A), and the bacteriocytes with butyrate esterase activity in symbiosomes were scattered in gill filaments (arrowheads in Fig. 4A). Although M6PR and mTORC1, as well as Rab9, LAMP1, and V-ATPase, were localized on the symbiosomes harboring symbionts, the symbionts gradually disappeared along with the symbiosomes (days 7 to 28 in Fig. 4A and fig. S3A). Chloroacetate esterase activity was also detected in the symbiosomes after 7 days of rearing and colocalized with butyrate esterase activity (fig. S4A). Esterase enzymes, such as lipase and phosphatase, assist in the intracellular digestion of bacteria (*36*, *37*). Our findings indicate that rearing under methane-depleted conditions triggered the phagosome digestion of symbionts and shifted the nutritional style from secretion by the symbionts to the digestion of symbionts.

Rapamycin, which inhibits the kinase activity of mTORC1, suppresses its downstream signaling pathway (the synthesis of proteins, lipids, nucleotides, and lysosome biogenesis) (*21*). To examine whether mTORC1 was involved in the phagosome digestion of symbionts, mussels were reared in a methane-depleted aquarium with or without rapamycin for 7, 14, and 28 days. During rearing with rapamycin, mTORC1 and M6PR, as well as Rab9, LAMP1, and V-ATPase, were detected on symbiosomes (Fig. 4B and fig. S3B). Butyrate and chloroacetate esterase activities were not detected in any bacteriocytes, and the symbionts remained in the bacteriocytes (Fig. 4B and fig. S4B). Interfering with the downstream signaling pathway of mTORC1 by rapamycin led to the suppression of the digesting symbionts with esterase enzymes and retention of the symbionts in symbiosomes even under methane-depleted conditions. When the mussels in the methane-depleted seawater were treated with rapamycin over a period of 14 days and then reared without rapamycin for 14 days (a total of 28 days of rearing), butyrate and chloroacetate esterase activities appeared in the symbiosomes harboring symbionts (arrowheads in Fig. 4C and fig. S4C), which were bound to mTORC1, M6PR, Rab9, LAMP1, and V-ATPase (Fig. 4C and fig. S3C). Cessation of rapamycin treatment led to the digestion of the symbionts with esterase enzymes. These results indicate that mTORC1 promoted the phagosome digestion of symbionts during rearing under methane-depleted conditions. In the transcriptome analysis using the mussels reared under methane-depleted conditions with or without rapamycin treatment, the expression levels of transcripts that we focused on as targets, such as Rab9, LAMP1, V-ATPase, M6PR, and mTORC1, did not show significant changes (fig. S5). These results corresponded to the detected signals observed in immunohistochemical analysis (Fig. 4, A and B, and fig. S3, A and B). In addition, there was no significant difference during rearing in the mRNA expression levels of genes listed in table S4, such as phagocytosis and mTORC1 pathway genes, regardless of the presence or absence of rapamycin (fig. S6A). Only a few genes showed significant expression differences during rearing, but there was no significant difference between the presence and absence of rapamycin (fig. S6B). This suggests that the rearing under methane-depleted conditions or the active state of mTORC1 has little effect on the expression of transcripts in these pathways. A previous study on T cells suggested that mTORC1 is involved in regulating M6PR transport (*38*), which delivers the synthesized lysosomal hydrolase to phagosomes (*7*, *34*). Although the detailed molecular interactions between mTORC1 and esterase enzyme activity have not been elucidated, the lack of esterase enzyme activity in symbiosomes harboring symbionts during rapamycin treatment may be due to the cessation of esterase enzyme production and/or M6PR transport to the symbiosomes having mTORC1.

### Promoting the phagosome digestion of exogenous dead symbionts by mTORC1

**Fig. 5.**
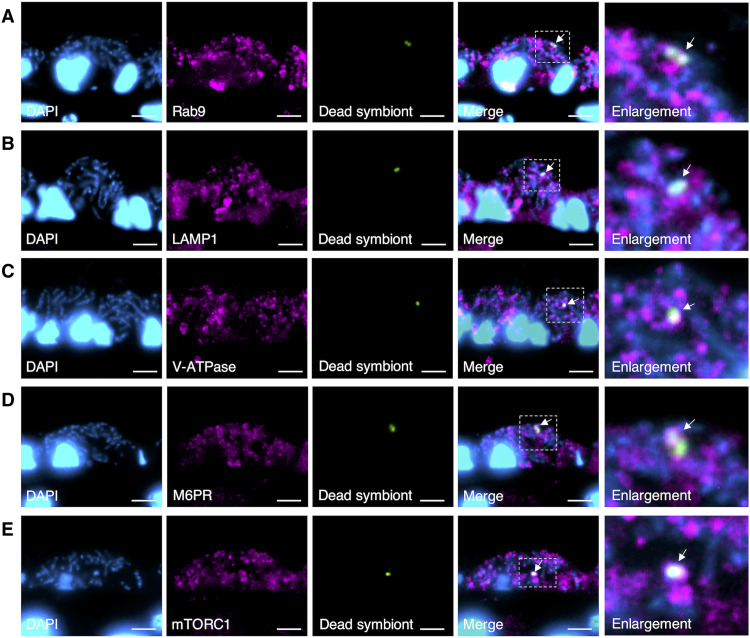
Immunohistochemistry of the phagosomes containing the exogenous dead symbionts (DSy). Immunofluorescence micrographs show fluorescence-labeled DSy (green), resident symbionts stained with DAPI (blue), and phagosomes stained with antibodies (magenta) against Rab9 (**A**), LAMP1 (**B**), V-ATPase (**C**), M6PR (**D**), and mTORC1 (**E**) in gill cells. The areas within the dotted square in the merged micrographs were magnified (Enlargement). Arrows indicate antibody-immunostained phagosomes with DSy in merged micrographs. Scale bars, 5 μm.

**Fig. 6.**
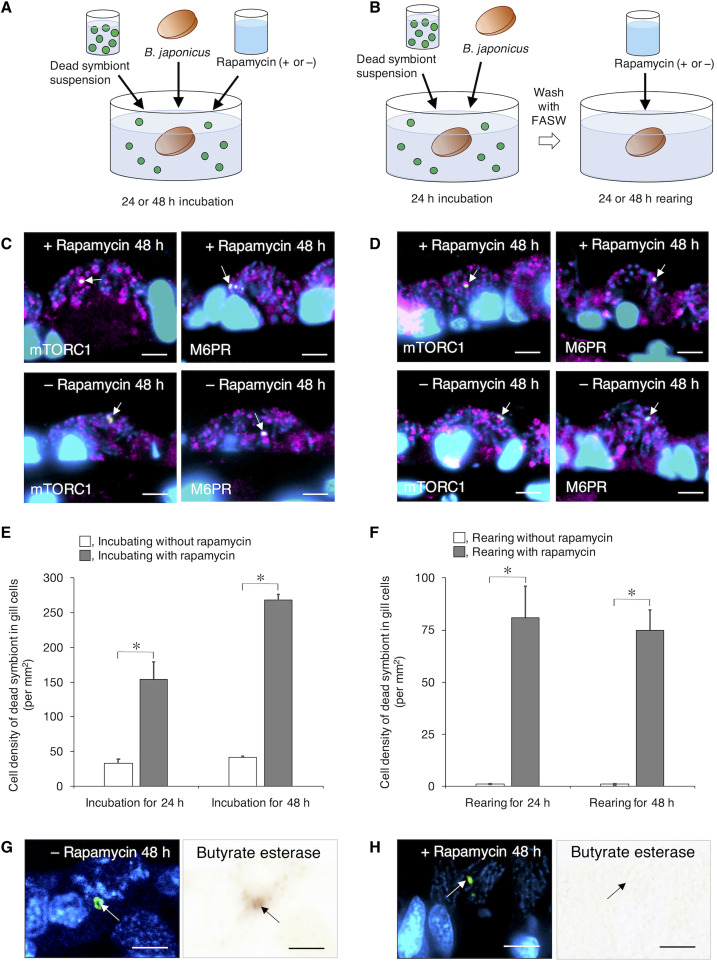
Immunohistochemistry and enzyme histochemistry of gill cells from the mussels incubated with exogenous dead symbionts and rapamycin. (**A**) Schematic depictions describe the experiments conducted on the mussels, which were incubated with dead symbionts in an aquarium with or without rapamycin for 24 or 48 hours. (**B**) Schematic depictions describe the experiments conducted on the mussels, which were initially incubated with dead symbionts for 24 hours and then with or without rapamycin for 24 or 48 hours. (**C**) Merged fluorescence micrographs show dead symbionts (green) in phagosomes bound to mTORC1 and M6PR (magenta) (arrows) from the experiment with (top) or without (bottom) rapamycin described in (A). (**D**) Dead symbionts were shown in phagosomes (arrows) from the experiment with (top) or without (bottom) rapamycin described in (B). (**E**) Comparison of cell densities (mean number ± per square millimeter of the sectioned gill cell area) of engulfed dead symbionts in gill cells [experiment of (A)]. (**F**) Comparison of cell densities (mean number per square millimeter) of engulfed dead symbionts in gill cells [experiment of (B)]. Gray bar, in the presence of rapamycin; white bar, in its absence. The cell densities of engulfed dead symbionts were determined by examining approximately 10 sections (each 2 μm thick). The data are expressed as means ± SD (per square millimeter of the examined gill sections) of three independent experiments (*P < 0.05, Student’s *t* test). (**G**) Merged fluorescence and bright-field micrographs show dead symbionts (green) in phagosomes with butyrate esterase (brownish red) from the experiment without rapamycin for 48 hours described in (A). (**H**) Merged fluorescence and bright-field micrographs show dead symbionts in phagosomes from the experiment with rapamycin for 48 hours described in (A). No butyrate esterase was detected in the bacteriocyte. Blue fluorescence, DAPI-stained host nuclei and resident symbiont cells. Scale bars, 5 μm.

In freshly collected mussel individuals, symbiosomes harboring symbionts in bacteriocytes showed no detectable esterase enzyme activity (day 0 in Fig. 4A and fig. S4A). In our previous study, when *B. japonicus* mussels were incubated for 24 hours with fluorescein isothiocyanate (FITC)–labeled dead symbionts extracted from the gills, the gill cells engulfed and preferentially digested them in vacuoles (phagosomes) with esterase enzymes. However, the resident symbionts in the symbiosomes remained intact without undergoing digestion in the same bacteriocyte (*13*). In the present study, after 24 hours of incubation, the vacuoles engulfing dead symbionts had Rab9, LAMP1, V-ATPase, M6PR, and mTORC1 (Fig. 5). This indicates that phagosomes containing dead symbionts were also characterized as phagolysosomes having mTORC1. If reducing the nutrient supply of the resident symbiont due to methane depletion induces mTORC1-mediated phagosome digestion, mTORC1 should also promote phagosome digestion of dead symbionts lacking nutrient production capability. To test this hypothesis, we conducted two different exposure experiments using dead symbionts under methane-depleted conditions with or without rapamycin: (i) mussels were incubated with dead symbionts with or without rapamycin for 24 or 48 hours (Fig. 6A), and (ii) mussels were first incubated with dead symbionts for 24 hours, washed, and then incubated with or without rapamycin for 24 or 48 hours (Fig. 6B). In both experiments, with or without rapamycin, mTORC1 and M6PR were detected on phagosomes containing exogenous dead symbionts (arrows in Fig. 6, C and D) and on symbiosomes harboring resident symbionts. In the incubation experiment with dead symbionts and rapamycin for 24 or 48 hours (Fig. 6A), the number of dead symbionts remaining in the phagosomes was significantly higher with rapamycin (gray bar in Fig. 6E) than in the absence of rapamycin (white bar in Fig. 6E; *t* test, *P* < 0.05). In the second experiment (Fig. 6B), dead symbionts were rarely found in the phagosomes of gill cells in the absence of rapamycin (white bar in Fig. 6F), whereas in the presence of rapamycin, significantly greater numbers of dead symbionts were found in phagosomes (gray bar in Fig. 6F; *t* test, *P* < 0.05). Rapamycin also suppressed the phagosome digestion of dead symbionts in the phagosomes of gill cells. During incubation of the mussels and dead symbionts with or without rapamycin for 48 hours (Fig. 6A), butyrate esterase activity was detected in the phagosomes containing dead symbionts in the absence of rapamycin during incubation (arrows in Fig. 6G) but not in any of the phagosomes in the presence of rapamycin (arrows in Fig. 6H). It did not appear to affect the protein expression of mTORC1 by rapamycin treatment. Instead, rapamycin might inhibit some factors associated with the initiation of phagosome digestion of dead symbionts with esterase enzymes by mTORC1. Therefore, these results suggest that mTORC1 promotes phagosome digestion of dead symbionts, which lack the ability to produce nutrients and supply them to the hosts.

### Controlling mTORC1-mediated phagosome digestion through nutrient signaling with symbionts

Generally, nutrient starvation in mammalian cells induces the dissociation of mTORC1 from lysosomes and its inactivation (*39*). In the present study, when the resident symbionts were enzymatically digested within symbiosomes in possibly starved mussels under methane-depleted conditions, mTORC1 was on the symbiosomes before and after the onset of phagosome digestion (Fig. 4A). In addition, one phagosome of a bacteriocyte that engulfed exogenous dead symbionts was shown to digest them by esterase; however, other surrounding symbiosomes harboring resident symbionts in the same bacteriocyte showed no trace of esterase; that is, no digestion of the symbionts was noted (Fig. 6G). These results suggest that mTORC1-mediated phagosome digestion is independently regulated in individual symbiosomes and phagosomes rather than by environmental or cytosolic factors. The bacterial pathogen *Salmonella* inhibits the translocation of Rab9 to late phagosomes by inhibitory factors excreted using type III secretion systems, thereby preventing M6PR transport and escape from intracellular digestion (*35*). While the symbionts in *B. japonicus* lack these secretion systems (*14*, *16*), they were stably harbored in the symbiosomes on which Rab9 and M6PR were found (Fig. 3, A and D) in fresh mussels before rearing (day 0 in Fig. 4A and fig. S3A). Meanwhile, when the symbiont was likely unable to produce any nutrients under methane-depleted conditions, the host mussel shifted its nutritional state from the symbiont retention mode to the digestion mode (Fig. 4A and fig. S4A). Considering that the dead symbiont could not produce nutrients and was selectively digested in the phagosomes (Fig. 6G), a deficient nutrient supply in the resident symbiont may trigger digestion.

**Fig. 7.**
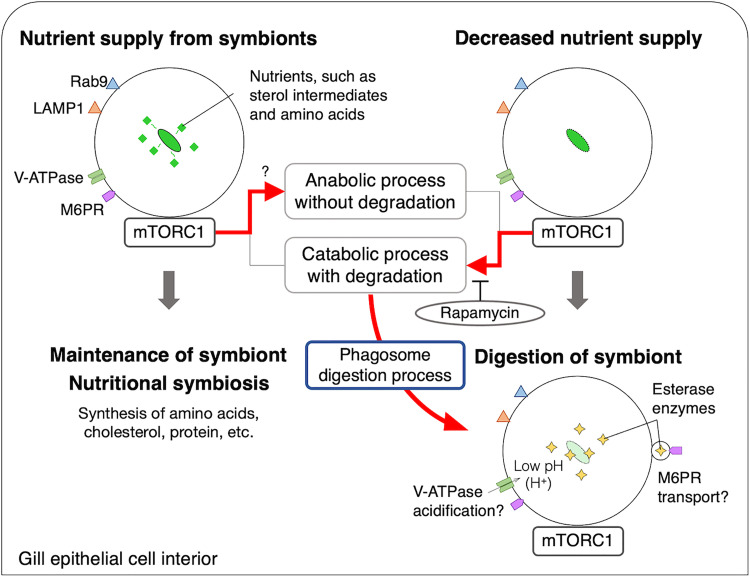
Schematic model for the regulation mechanism of mTORC1-mediated phagosome digestion through nutrient signaling with symbionts in *B. japonicus*. mTORC1 localizes to symbiosomes harboring symbionts that also have Rab9, LAMP1, V-ATPase, and M6PR. Nutrient supply from the symbiont controls mTORC1-mediated phagosome digestion. In this case, the host mussel most likely promotes anabolic metabolism in the mTORC1 pathway by using nutrients from the symbionts. When the nutrient supply from symbionts is reduced, mTORC1 promotes catabolic metabolism with degradation and digests the symbionts by esterase enzymes. In this case, the interior of the symbiosome may be acidified by the action of V-ATPase (*13*), and the esterase enzyme produced in the gill cell may be transported into the symbiosome via M6PR. This digestion process was inhibited by rapamycin.

mTORC1 receives signals from nutrients such as cholesterol and amino acids inside lysosomes via signaling components such as V-ATPase and solute carrier family 38 member 9 (SLC38A9), which are lysosomal transmembrane proteins associated with Ragulator-Rag guanosine triphosphatase complexes (*32*, *33*, *40*). Upon receiving these signals, mTORC1 promotes either protein synthesis or degradation in response to the nutritional state of the cell (*22*, *24*, *32*, *33*). SLC38A9 and V-ATPase were found in the gills of *B. japonicus* using transcriptome analysis (table S4). It has been suggested that an adequate nutrient supply from symbiotic microbes to hosts is closely associated with establishing symbiosis (*41*–*43*). In addition, a previous genomic study on aphid-*Buchnera* symbiosis suggested that the mTOR pathway is involved in the nutritional interaction between the host and symbiont (*44*). In our present stable isotope analysis results, at least before the onset of rearing, the symbiont produced some nutrients for the host mussel. The host received methane-derived carbon compounds, such as steroids and amino acid intermediates, from the symbiont (fig. S1, C and D, and Fig. 2A). Our findings suggest that mTORC1 regulates phagosome digestion in response to the nutrient supply from the symbionts inside each symbiosome. mTORC1 on lysosomes is known to coordinate cellular anabolic and catabolic metabolism to maintain cell homeostasis (*21*–*24*). If the nutrients themselves and/or unknown blocking factors from the symbionts suppress the activity of mTORC1, then the function of mTORC1 as a regulator of host cellular metabolism would be lost in bacteriocytes along with inhibiting phagosome digestion. Therefore, in host bacteriocytes, mTORC1 may be involved in both regulations of biosynthesis using nutrients from symbionts and the regulation of phagosome digestion of symbionts. We propose a model for the maintenance mechanism of symbionts in *Bathymodiolus* mussels through cross-talk by mTORC1 between nutrient signaling and the phagocytic process (Fig. 7). In natural habitats, when the symbionts supply optimal nutrients to the host mussels in symbiosomes, sensing their nutrients leads the host to the cessation of mTORC1-mediated phagosome digestion of the symbiont. mTORC1 may promote intracellular metabolism without digestion through probable nutrient signaling with the symbionts, thereby sustaining intracellular nutritional symbiosis in the host mussel. In contrast, if the nutrient supply from symbionts is reduced due to the depletion of energy and carbon sources, mTORC1 promotes phagosome digestion of the symbionts with reduced nutrient supply.

### Perspective and limitation

The present study demonstrates that controlling mTORC1-mediated phagosome digestion is critical for maintaining symbionts in *Bathymodiolus* mussels. Symbiosomes with mTORC1, characterized as phagolysosomes, act as selection sites for retaining or digesting symbionts and eliminating other bacteria by regulating phagosome digestion and nutrient signaling. The host mussel allows it to retain its symbiont through nutrient sensing even if the symbiont has no blocking factors for survival in phagosomes. In contrast, a decrease in the production and supply of nutrients from the symbiont to bacteriocytes triggers the promotion of mTORC1-mediated phagosome digestion of the symbiont. These findings suggest that nutrient signaling from symbionts to mTORC1 in the symbiosomes may be a crucial mechanism underlying the intracellular symbiosis of *B. japonicus*. It is necessary to identify the nutrients from the symbionts and their metabolites in bacteriocytes to understand this mechanism in the mussel. However, it is difficult because symbiont pure cultivation and mussel artificial rearing have not been established. By focusing on the digestion of the symbionts through mTORC1 and phagocytic pathways in mussels under methane-depleted conditions, a comprehensive analysis is necessary to identify the factors that switch between the retention and digestion of the symbionts in the future. It is interesting to study whether the mTORC1 pathway also functions to maintain the symbionts in other *Bathymodiolus* mussels. It is also noteworthy that once digestion in the symbiosome and phagosome is initiated, nutrients should be released from the digested bacteria; however, the digestion process did not cease even though mTORC1 and other examined components remained on the symbiosome and phagosome (Figs. 4 and 6). This suggests that once the digestion process is initiated, it is irreversible and cannot be restored to the symbiosome. After digestion of the symbiont, the symbiosome-related factors, including mTORC1 and M6PR, disappeared from the bacteriocytes. Furthermore, previous studies in the *Bathymodiolus* mussels have reported that the cellular remodeling in gills with apoptosis is caused by a long-term rearing under energy-depleted conditions for the symbionts (*45*, *46*). mTORC1 is also known to be involved in autophagy, cell growth, and proliferation (*24*). It may be important in the maintenance and clearance of gill cells and symbionts by coordinating apoptosis and cell proliferation. It is necessary to study the involvement of mTORC1 in such cellular functions in the future. Our findings suggest that mTORC1 allows the selective maintenance of symbionts by integrating the cellular functions of feeding, immune defense, and nutrient signaling through interactions between host and microbe. mTORC1 is highly conserved among eukaryotes as a central regulator of nutrient metabolism in lysosomes. It may be a potential factor contributing to the evolution and development of various host-microbe intracellular symbiosis. Understanding the regulation of phagocytosis by mTORC1 would help to elucidate the nexus animal-microbe and provide deeper insights into the underlying establishment and maintenance mechanism of intracellular nutritional symbiosis.

## MATERIALS AND METHODS

### Collection of *B. japonicus*

*B. japonicus* was collected at the Hatsushima Island seep site in Sagami Bay, Japan, using the remotely operated vehicle (ROV) Hyper-Dolphin and Kaimei (KM)-ROV (Hyper-Dolphin dive #1076, #1126, #1284, #1992, and #2098 to 2099; KM-ROV dive #111 to 112 and #159; and the deep submergence vehicle Shinkai 6500 dive #1557 ; 35°00.919′ to 35°00.966′N:139°13.329′ to 139°13.433′E; depth: 873 to 978 m) during cruises of the Research Vessel (RV) *Natsushima*, RV *Shinseimaru*, RV *Kaimei*, RV *Yokosuka* (NT10-01, 12 to 18 January 2010; NT10-08, 11 to 17 May 2010; NT11-09, 15 to 26 June 2011; KM16-04, 30 June to 5 July 2016; KS-20-1, 7 to 11 January 2020; KM20-09, 14 to 22 November 2020; KM21-08, 23 to 25 October 2021; and YK19-11, 28 August to 14 September 2019). The water temperature, salinity, and dissolved oxygen measurements were 2.8° to 4.1°C, 34.3 to 34.6, and 1.1 to 1.3 ml/liter, respectively. The mussels were immediately transferred to the shipboard laboratory and maintained in tanks containing 60 liters of 0.22 μm–filtered natural seawater at 4° to 5°C and a salinity of 35‰. For in situ RNA stabilization of *B. japonicus*, including its methane-oxidizing symbiotic bacteria in transcriptome analysis, the mussels whose shells were cracked by the manipulator of the ROV were preserved in RNAlater in situ at their habitat using a previously described method (*47*). The experiments were conducted and approved by the ethics committee in Japan Agency for Marine-Earth Science and Technology (experimental ID: R04-02) in accordance with the Guidelines for Proper Conduct of Animal Experiments (Science Council of Japan).

### Dual transcriptome sequencing

Total RNA from the gills of one *B. japonicus* fixed in situ and mussels after rearing (described below) was extracted using NucleoSpin RNA (MACHEREY-NAGEL Inc.). The quantity and quality of RNA were measured using the Qubit RNA High Sensitivity Assay Kit with a Qubit 1.0 Fluorometer (Invitrogen) and the Agilent RNA 6000 Pico Kit with an Agilent 2100 Bioanalyzer (Agilent). The RNA samples were placed at −80°C until sequencing. Total RNA was progressively treated with the RiboMinus Eukaryote System version 2 (Thermo Fisher Scientific) and the Ribo-Zero rRNA Removal Kit for Gram-negative bacteria (Epicentre) to prepare ribosomal RNA (rRNA)–depleted RNA. Strand-specific RNA-seq libraries were constructed by complementary DNA synthesis using the SMARTer Stranded RNA-Seq Kit (Clontech), fragmentation with a Covaris M220 instrument (Covaris), and adaptor ligation with the KAPA Hyper Prep Kit (KAPA Biosystems). The libraries obtained were sequenced on the Illumina HiSeq2500 platform (Illumina) at Macrogen Inc., to yield 276 M reads of paired-end reads (250 bp). Raw sequencing data of the *B. japonicus* transcriptome have been deposited in the National Center for Biotechnology Information (NCBI) Sequence Read Archive database under the accession number DRR216663.

De novo dual transcriptome assembly and sequence analysis of *B. japonicus* were performed with RNA-seq data from an in situ fixed sample. Raw reads were cleaned using the Trimmomatic version 0.36 (*48*) to remove the adapter and low-quality sequences. The reads were further filtered with the Preprocessing and Information of Sequence data (PRINSEQ) tool (*49*) for removal of duplicate reads and low-complexity homopolymeric reads using “-lc_method dust -lc_threshold 32.” The remaining reads were assembled using Trinity version 2.0.6 (*50*) with default parameters. All transcript sequences were further processed to form nonredundant transcript redundancy using the EvidentialGene tr2aacds pipeline (2013, http://arthropods.eugenes.org/EvidentialGene/). The pipeline selects the “best” set of de novo assembled transcripts based on coding potential. Protein-coding regions were predicted using the TransDecoder program (*51*), and their functional annotation was performed by homology searches against the National Center for Biotechnology Information (NCBI) nonredundant (nr) database and Kyoto Encyclopedia of Genes and Genomes protein databases (*52*) with a threshold E-value of 1 × 10^−5^. The protein-coding transcripts were selected and aligned against the nr database using DIAMOND (version 0.9.17) in the BLASTx mode and setting a threshold E-value of 1 × 10^−5^ (*53*). Taxonomic assignment in protein-coding transcripts was conducted using Metagenome Analyzer MEGAN6, setting a minimum score of 50 and a cutoff of the top 10% using the alignment results (*54*). BUSCO version 5.4.5 was used to evaluate the comprehensiveness of the *B. japonicus* and symbiont transcriptome assemblies (*55*). Gene expression was calculated through RNA-seq by an Expectation-Maximization simulator, RNA-Seq by Expectation Maximization version 1.2.23 (*56*) using Bowtie2 version 2.2.5 (*57*). Differential expression analysis was performed by the DESeq2 R package (version 3.17). Differential expression genes were defined by the adjusted *P* value below 0.01 between rearing days.

### Stable isotopic analysis

Stable carbon and nitrogen isotopic analyses of the bulk gill and foot (*n* = 3) were performed to identify the mussels’ nutrient sources before rearing. We used amino acid nitrogen isotopic analysis to estimate the amino acid metabolism of mussels during aquarium rearing in methane-depleted conditions (*28*). After collection, the mussels (60 to 78 mm shell length) were reared in onshore tanks with 0.22 μm–filtered artificial seawater (FASW; 3.5% Rohto Marine; Rei-Sea) using tap water for 0, 28, and 485 days at 4° to 5°C. After rearing, the gill, foot, and mantle were excised from each mussel, immediately frozen, and stored at −80°C. For fluorescence microscopy, gill pieces from each of the three individuals were excised and fixed with 4% paraformaldehyde in FASW. For stable carbon and nitrogen isotopic analyses of bulk tissues, frozen gills and foot before rearing were analyzed using a previously described method (*58*). For nitrogen isotope analysis of amino acids, the isotopic compositions of the gill, foot, and mantle of individuals reared for 0, 28, and 485 days were analyzed as described previously (*28*, *59*). The TP of the specimens was also calculated on the basis of the nitrogen isotopic composition of glutamic acid (δ^15^N_Glu_) and phenylalanine (δ^15^N_Phe_) using the formula TP_Gln/Phe_ = (δ^15^N_Glu_ − δ^15^N_Phe_ − 3.4)/7.6 + 1, as described previously (*28*).

### Rearing experiments of the mussels

Three rearing experiments were conducted after the sampling. In the first rearing experiment, the mussels (60 to 78 mm shell length) were reared in tanks with FASW without any chemical components, such as methane, for 0, 7, 14, 28, and 485 days at 4° to 5°C. Considering that nitrogen metabolism in the mussels was expected to be very slow, the mussels reared for 485 days were used as samples, the protein of most of which were expected to be replaced with the newly synthesized protein after rearing. In the second rearing experiment, to test the effect of suppression of mTORC1activity on the disappearance of symbionts during rearing, the mussels were reared with rapamycin (LC Laboratories Inc.) at a final concentration of 2.8 μM in FASW for 7, 14, and 28 days at 4° to 5°C. Rapamycin was added to FASW in a tank with mussels every 48 hours, along with FASW exchange during the rearing. In the third experiment, after the mussels were reared in FASW with rapamycin (final concentration of 2.8 μM) for 14 days, they were rinsed with FASW and then reared in FASW without rapamycin for 14 days at 4° to 5°C. The mussels were reared in a single tank and sampled on specified days in these rearing experiments. After each rearing period, the gill pieces from the three or four individuals were excised and fixed with 4% paraformaldehyde in FASW and also preserved in RNAlater in the first and second experiments. For immunohistochemistry, samples for 0, 7, 14, and 28 days in these rearing experiments were examined. For transcriptome analysis, samples for 0, 7, 14 days were examined in the first and second rearing experiments.

### Incubation of the mussels with fluorescence-labeled dead symbionts

Methane-oxidizing symbionts were extracted from the gills of *B. japonicus* as described previously (*60*). The symbionts were fixed with 4% paraformaldehyde in FASW for 24 hours at 4°C. The dead symbionts were labeled with FITC, as described previously (*13*). The small-sized mussels (20 to 29 mm shell length) were placed in a 200 ml container with 150 ml of FASW and individually incubated with dead symbionts (final density of 1 × 10^6^ to 10 × 10^6^ cells/ml) in darkness for 24 and 48 hours at 4°C. To test the effect of suppression of mTORC1 activity on the phagosome digestion, the mussels were incubated with dead symbionts in FASW containing 2.8 μM rapamycin for 24 and 48 hours at 4°C in darkness. In addition, the mussels were incubated with dead symbionts for 24 hours at 4°C in darkness, then washed with FASW, and incubated in the presence or absence of rapamycin in FASW for 24 and 48 hours at 4°C in darkness. After the incubation, each mussel was rinsed with FASW; the gills were excised using scalpels, cleaned with alcohol, and fixed with 4% paraformaldehyde in FASW for at least 24 hours at 4°C.

### Preparation of semithin sections

Following procedures from previously published protocols (*13*), we prepared semithin sections. Briefly, the gill pieces fixed with 4% paraformaldehyde in FASW were washed with FASW, dehydrated in a graded series of ethanol (30, 50, 70, 90, and 100%), and embedded in Technovit 8100 resin (Heraeus Kulzer) at 4°C. Semithin sections (2 μm thick) were cut using a glass knife mounted on an Ultracut S ultramicrotome (Leica Microsystems, Wetzlar, Germany) and collected on S9445 glass slides (Matsunami Glass, Osaka, Japan).

### Fluorescence in situ hybridization

To detect methane-oxidizing symbiotic bacteria in bacteriocytes of *B. japonicus* on rearing day 0, which were stained with DAPI, HCR-FISH of the gill sections from mussels on rearing day 0 was performed as described previously (*61*, *62*) with the following modifications. The sections were washed in phosphate-buffered saline (PBS) and hybridized at 46°C using a solution containing 20% formamide, 0.9 M NaCl, 20 mM tris-HCl (pH 7.5), 10% dextran sulfate, 1% blocking reagent (Roche), 0.01% SDS, and 0.5 μM initiator probe (5′-CCGAATACAAAGCATCAACGACTAGAAAAAAGTTAGCTCCGCCACTAAACCTGT-3′), which was designed to be specific for the 16*S* rRNA of some Methylococcales bacteria, including the *B. japonicus* symbiont. After hybridization, the excess probe was washed twice with a solution containing 0.225 M NaCl, 20 mM tris-HCl (pH 7.5), and 0.01% SDS for 15 min at 48°C. During washing, 5 μM H1 and H2 amplifier probes (H1, 5′-TCTAGTCGTTGATGCTTTGTATTCGGCGACAGATAACCGAATACAAAGCATC-3′; and H2, 5′-CCGAATACAAAGCATCAACGACTAGAGATGCTTTGTATTCGGTTATCTGTCG-3′) labeled with Alexa Fluor 488 were incubated independently in the amplification buffer (50 mM Na_2_HPO_4_, 0.9 M NaCl, 10% dextran sulfate, and 0.01% SDS) for 1.5 min at 95°C and 30 min at 25°C. Subsequently, the H1 and H2 amplifier probes were mixed, and 10 μl of the amplifier probe mixture was applied to the sections. The sections were incubated for 2 hours at 46°C in a humidified chamber and washed with PBS for 10 min at 4°C. To confirm that the symbionts detected by HCR-FISH were the same as those stained with DAPI, the sections were stained with DAPI (2 μg/ml in distilled water) and observed using a fluorescence microscope with UV-1A [excitation (Ex) wavelength, 345 to 365 nm; emission (Em) wavelength > 400 nm] and FITC (Ex, 465 to 495 nm; Em, 515 to 555 nm).

### Electron microscopy

To observe the localization of symbionts within symbiosomes in bacteriocytes, the whole-mount small mussels (shell length of about 7 mm) were fixed with 2.5% glutaraldehyde in FASW for at least 24 hours at 4°C. After fixation, they were washed with FASW, postfixed with 2% osmium tetroxide in FASW for 2 hours at 4°C, conductively stained with 0.5% thiocarbohydrazide (Thermo Fisher Scientific) aqueous solution for 30 min and with 1.0% osmium tetroxide aqueous solution for 1 hour at 4°C, dehydrated in a graded series of ethanol (30, 50, 70, 90, and 100%), and embedded in Epon 812 (TAAB laboratories equipment ltd., Aldermaston, UK) at 65°C. Semithin sections (500 nm) were cut using a glass knife mounted on an Ultracut S ultramicrotome (Leica Microsystems), stained with 2% uranyl acetate and 2% lead citrate solutions, and observed using a Quanta 450 field-emission gun (FEG) field-emission scanning electron microscope (SEM) (FEI company) with a backscattered electron detector operating at 4 kV. The semithin sections were also stained with aqueous 0.1% toluidine blue at approximately 80°C for 1 min and imaged using the Optiphot light microscope.

In TEM, after fixation with glutaraldehyde of the gills from the mussel reared on days 0, 28, and 485, the gills were postfixed by incubating for 2 hours at 4°C in 2.0% osmium tetroxide in FASW, dehydrated in a graded series of ethanol, and embedded in Epon 812 resin (Nisshin EM) at 60°C. Ultrathin sections (70 nm thick) were cut using a diamond knife mounted on an Ultracut S ultramicrotome (Leica Microsystems, Wetzlar, Germany) and stained with a 2.0% uranyl acetate solution and a 2.0% lead citrate solution. The sections were observed using a Tecnai G2 20 electron microscope (FEI) operated at 200 kV.

### Polyclonal antibodies

Polyclonal antibodies were generated in rabbits using a synthetic peptide as an antigen (Cosmo Bio Inc.). Peptides corresponding to the cation-independent M6PR (amino acid position 2447 to 2467, CILNPFEDAGGYHDDSDEDLLA), mTORC1(amino acid position 1695 to 1712, CKALQLISPEDSQQRQDLN), LAMP1 (amino acid position 130 to 147, CHQAYNADQFYALTSGSYR), Rab9 (amino acid position 165 to 181, CFKAAIRRIRELEDVIEM), and V-ATPase subunit F (amino acid position 2447 to 2467, CNTPRHDIEEAFRGFL) from *B. japonicus* were selected using epitope estimation in-house software (Cosmo Bio Inc.) and synthesized. These peptides were conjugated to ovalbumin through cysteine at the N terminus of these peptides and injected into rabbits. Booster injections were administered to each rabbit on days 14, 28, and 42. Antisera were obtained by bleeding the animals on day 56. The antibodies were purified by affinity to an antigen-conjugated column and used for immunoblotting to test their specificity.

### Immunohistochemistry

Gill sections (2 μm thick) embedded using Technovit 8100 were treated with 2% block ace (KAC Ltd.) in 1 × PBS for 30 min at approximately 23°C. They were then incubated with the polyclonal antibodies against Rab9, LAMP1, V-ATPase, M6PR, and mTORC1 diluted 1:200 in PBS for 12 hours at 37°C. After incubation, the sections were incubated with CF565-conjugated goat anti-rabbit immunoglobulin G (IgG) secondary antibody (1:200 dilution in PBS; Nacalai Tesque) for 2 hours at about 23°C, stained with DAPI, and observed under a BX-51 light and fluorescence microscope (Olympus, Tokyo, Japan) with UV (Ex, 330 to 385 nm; Em, >400 nm), FITC (Ex, 470 to 495 nm; Em, 510 to 550 nm), and Cyanine 3 (Cy3) (Ex, 530 to 570 nm; Em, 573 to 648 nm) filter sets for DAPI, antibodies, and fluorescence-labeled dead symbionts, respectively. For immunoelectron microscopy using SEM, the gill sections from each reared day’s sample were incubated with five polyclonal antibodies as described above and subsequently incubated with 20 nm gold particles conjugated to goat anti-rabbit IgG secondary antibody (1:100 dilution in PBS; Sigma-Aldrich) for 2 hours at approximately 23°C. The sections were postfixed with 1% glutaraldehyde in PBS, stained with 2% uranyl acetate solution, coated with osmium, and observed using a Quanta 450 FEG field-emission SEM with a backscattered electron detector operating at 4 kV.

### Histochemical detection of butyrate esterase activity

Semithin sections (2 μm thick) of gill pieces from the reared mussels and the incubated mussels with dead symbionts were incubated for 12 hours at about 23°C with α-naphthyl butyrate solution and fast garnet GBC base prepared using an esterase staining kit (Muto Pure Chemical) according to the manufacturer’s instructions. Butyrate esterase activity was observed as a brownish-red color. As negative controls, the sections were incubated for 12 hours at about 23°C with a solvent dye solution of fast garnet GBC base without adding the substrates of α-naphthyl butyrate. For double staining with immunohistochemistry, after immunological staining of the sections from the reared mussels with anti-M6PR and mTORC1 antibodies, butyrate esterase activity in the sections was detected as described above. The sections were then stained with DAPI for 5 min at about 23°C, air-dried, mounted with Vectashield, and covered with a coverslip. The sections were imaged using light and fluorescence microscopy with UV, FITC, and Cy3 filter sets for DAPI, fluorescence-labeled dead symbionts, and antibodies, respectively.

The semithin sections were also incubated for 12 hours at about 23°C with naphthol AS-D chloroacetate solution and fast blue RR salt prepared using an esterase AS-D assay kit (Muto Pure Chemical) according to the manufacturer’s instructions. Chloroacetate esterase activity was observed as a blue color. Double immunohistochemical staining was not performed because chloroacetate esterase staining quenched the fluorescence signals in the secondary antibody and DAPI. For double staining of butyrate and chloroacetate esterase, the sections were washed with distilled water, stained as described above, mounted with glycerol gelatin, and imaged using a light microscope.

### Statistical analysis

Statistical analyses were performed using Microsoft Office 2016 Excel for Mac (version 16.16.27, Microsoft Corporation). To compare cell densities of fluorescence-labeled dead symbionts in gill cells in the presence or absence of rapamycin treatment, internalized dead symbionts were counted in 30 gill sections per mussel individual under each experimental condition for a total of 12 mussel individuals. Student’s *t* test was carried out to determine whether the cell densities of dead symbionts in the gill cells differed significantly depending on the presence or absence of rapamycin during incubation with dead symbionts. Statistical significance was set at *P* < 0.05.
