# Supplementary material for: mTORC1 regulates phagosome digestion of symbiotic bacteria for intracellular nutritional symbiosis in a deep-sea mussel
Source: Sci Adv. 2023 Aug 23;9(34):eadg8364. doi: 10.1126/sciadv.adg8364 (PMC10446485; doi:10.1126/sciadv.adg8364)
Supplement: Supplementary file 1 — Figs. S1 to S6 Legends for tables S1 to S5 [file sciadv.adg8364_sm.pdf]

Science Advances
AAAS

Supplementary Materials for

mTORC1 regulates phagosome digestion of symbiotic bacteria for intracellular nutritional symbiosis in a deep-sea mussel

Akihiro Tame *et al.*

Corresponding author: Takao Yoshida, tyoshida@jamstec.go.jp

Sci. Adv. **9**, eadg8364 (2023)
DOI: 10.1126/sciadv.adg8364

The PDF file includes:

Figs. S1 to S6
Legends for tables S1 to S5

Other Supplementary Materials for this manuscript includes the following:

Tables S1 to S5

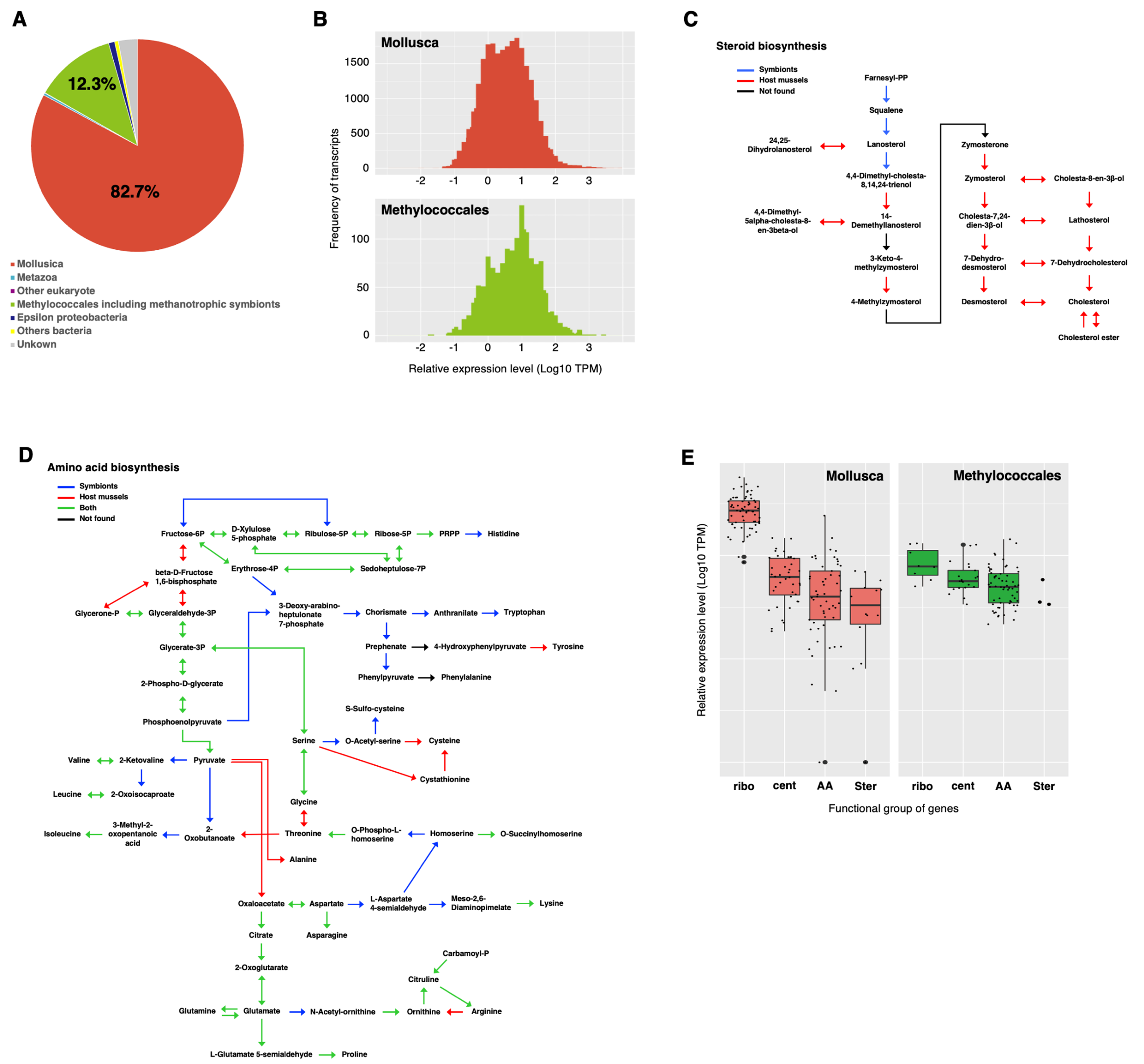

Fig. S1. Comparison of transcriptomes of the host mussel, *Bathymodiolus japonicus*, and its symbionts. (**A**) The circle graph shows the taxonomic proportions of the reads mapped to each transcript. (**B**) The histograms show the relative expression levels of transcripts in each Mollusc to which *Bathymodiolus* mussels belong (top) and Methylococcales to which methane-oxidizing symbionts belong (bottom). (**C**) A scheme for cholesterol biosynthetic pathway shows the genes found in the mussel and symbiont based on the Kyoto Encyclopedia of Genes and Genomes (KEGG) database. Red and blue lines indicate genes found in the mussel and symbiont, respectively. Black lines indicate the estimated reaction not found in either the mussel or symbiont genome. (**D**) Based on the KEGG database, a scheme for amino acid biosynthetic pathway shows the genes found in the mussel and symbiont. Red, blue, and green lines indicate genes found in the mussel, symbiont and both. (**E**) Relative expression levels of metabolism- and biosynthesis-related genes in the hosts and symbionts. The light red boxplot and light green boxplot indicate relative expression levels in transcripts per million (TPM) in the mussel and symbiont, respectively. Ribosomal proteins (ribo), central metabolism (cent) included glycolysis, the tricarboxylic acid cycle, and the pentose-phosphate cycle, as well as biosynthesis of amino acid (AA) and steroid (Ster).

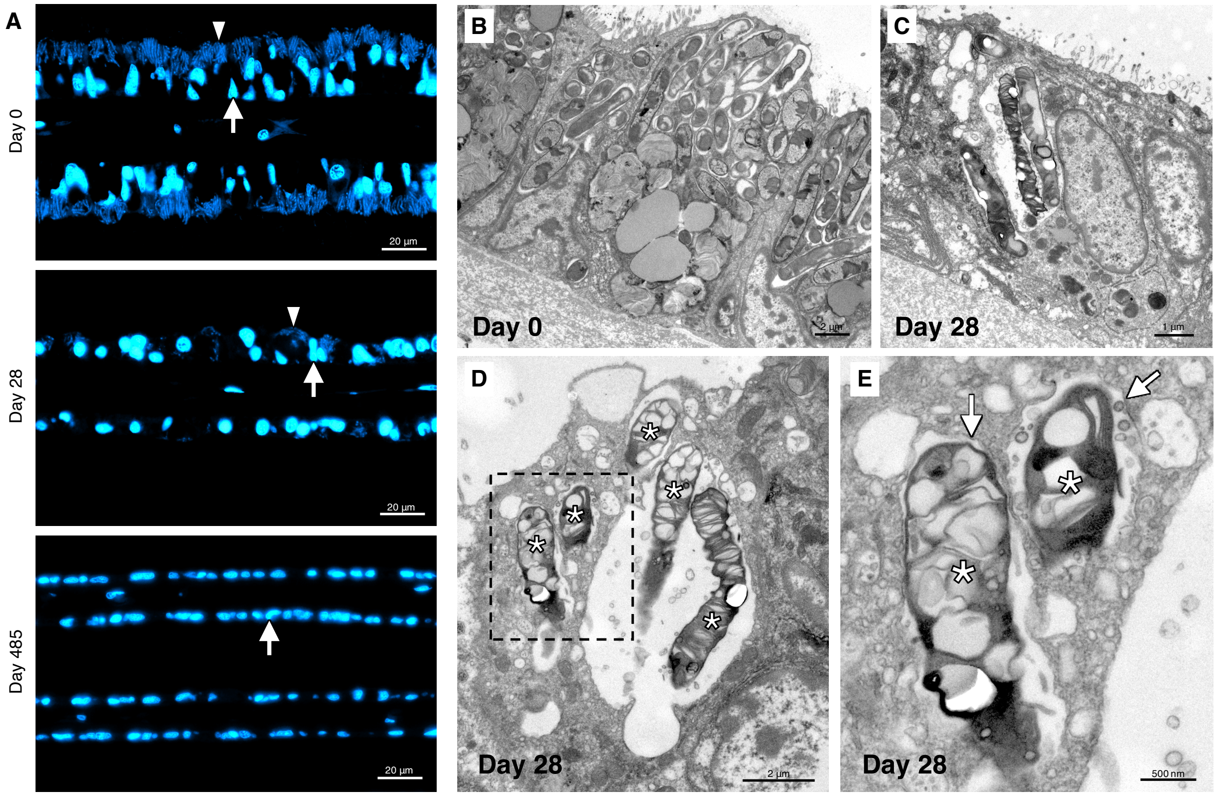

Fig. S2. Disappearance of symbionts from the bacteriocytes during mussels rearing. (**A**) Fluorescence micrographs of gill sections stained with DAPI (blue) show the decrease of symbionts from bacteriocytes during mussel rearing in the absence of energy and carbon source for symbionts at rearing days 0, 28, and 485. Scale bars, 20 µm. Arrows indicate the nuclei of host mussels stained with DAPI. Arrowheads indicate the symbionts stained with DAPI. (**B**) TEM micrograph shows the bacteriocytes of mussels before the rearing. (**C**) TEM micrograph shows the bacteriocytes of mussels rearing for 28 days under a methane-depleted condition. (**D**) TEM micrographs show the rod-shaped symbionts having unclear cell surfaces and inside components in bacteriocytes of mussels after rearing on day 28. (**E**) High magnification of the TEM micrograph shows the symbionts in dashed-square in **D**. Asterisks indicate the symbionts. Arrows indicate the single-membrane symbiosomes.

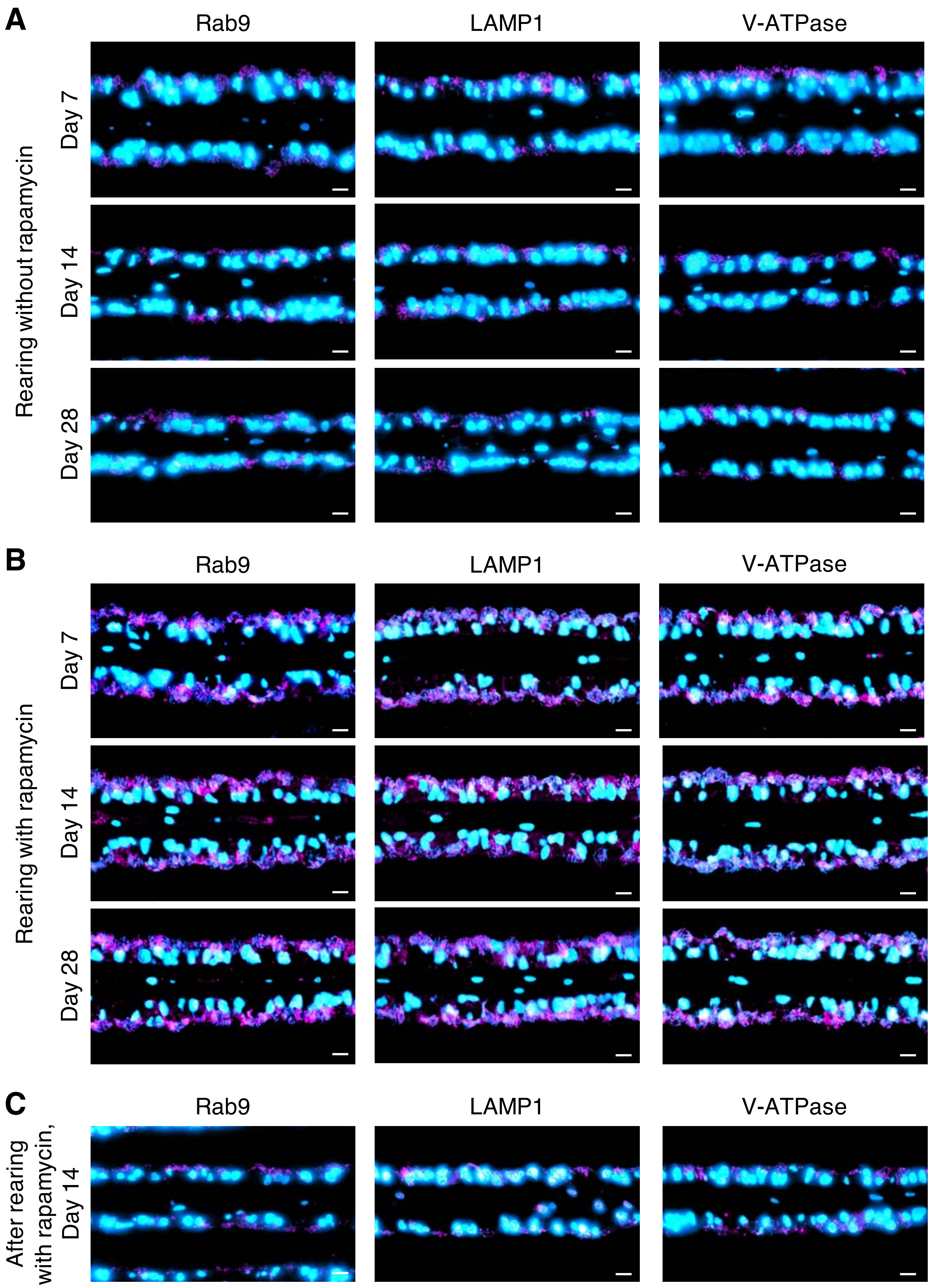
A
Rab9
LAMP1
V-ATPase
Rearing without rapamycin
Day 7
Day 14
Day 28
B
Rab9
LAMP1
V-ATPase
Rearing with rapamycin
Day 7
Day 14
Day 28
C
Rab9
LAMP1
V-ATPase
After rearing with rapamycin, Day 14

Fig. S3. Immunohistochemistry of gill cells from the mussels reared with or without rapamycin. (**A**) Mussels were reared without rapamycin under a methane-depleted condition for 7, 14, and 28 days. Merged fluorescence micrographs of DAPI (blue) stained symbiont cells in symbiosomes, which were concomitantly stained with antibodies (magenta) against Rab9 (left), LAMP1 (middle) or V-ATPase (right) in gill sections. After day 7 of rearing, the DAPI-stained symbiont began to disappear along with the symbiosomes bound to Rab9, LAMP1, or V-ATPase. (**B**) Mussels were reared with 2.8 μM rapamycin under a methane-depleted condition for 7, 14, and 28 days. Merged fluorescence micrographs of symbiosomes with DAPI-stained symbionts and those stained with antibodies (magenta) against Rab9 (left), LAMP1 (middle), or V-ATPase (right) in gill sections. The resident symbionts were retained in the symbiosomes bound to Rab9, LAMP1, or V-ATPase during rearing with rapamycin. (**C**) Mussels were reared with rapamycin under methane-depleted conditions for 14 days and then without rapamycin for 14 days. Merged fluorescence micrographs show the sectioned gill epithelia stained with DAPI and with antibodies against Rab9 (left), LAMP1 (middle), or V-ATPase (right). Scale bars, 10 μm.

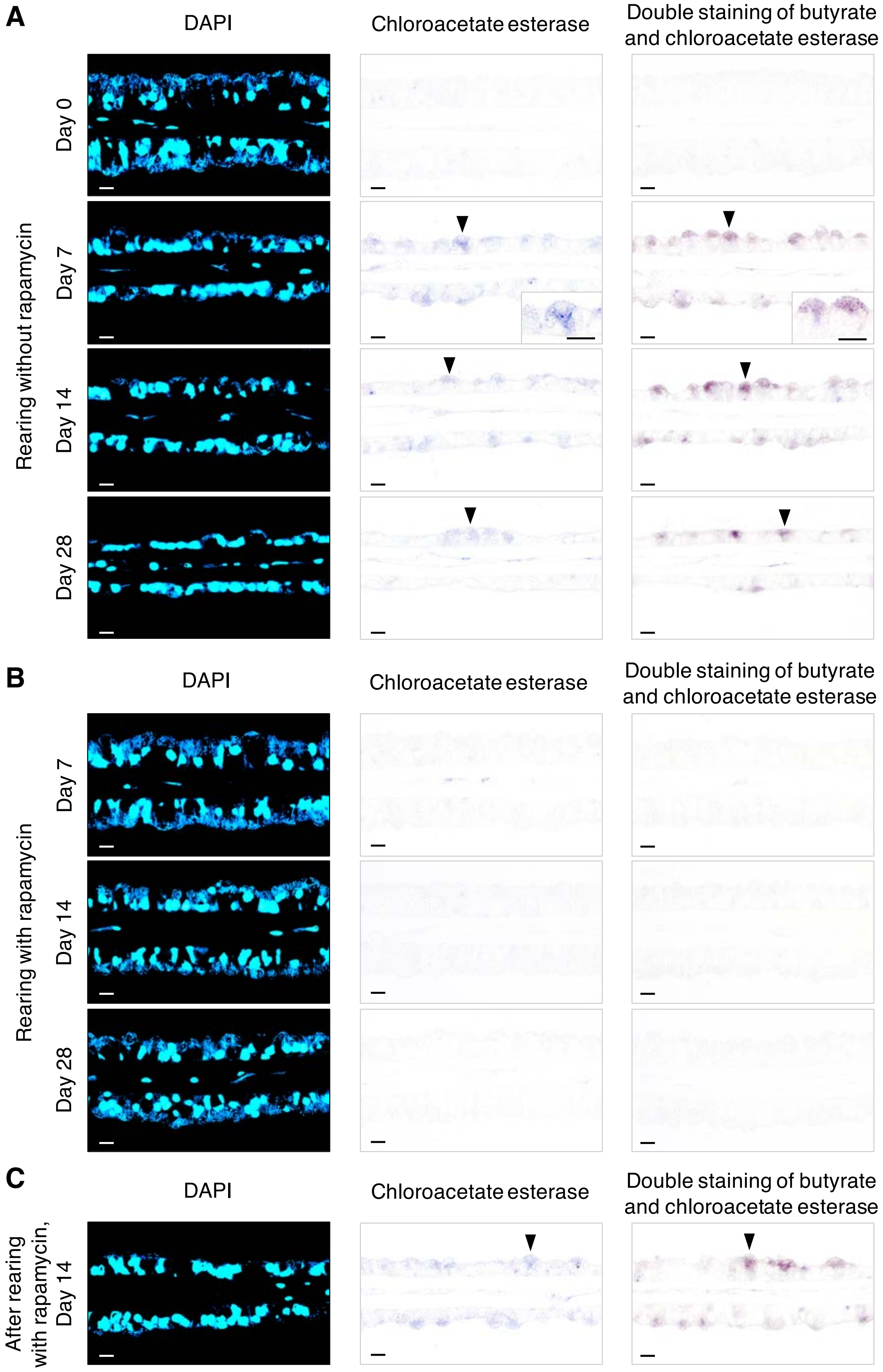
A
DAPI
Chloroacetate esterase
Double staining of butyrate and chloroacetate esterase
Rearing without rapamycin
Day 0
Day 7
Day 14
Day 28
B
DAPI
Chloroacetate esterase
Double staining of butyrate and chloroacetate esterase
Rearing with rapamycin
Day 7
Day 14
Day 28
C
DAPI
Chloroacetate esterase
Double staining of butyrate and chloroacetate esterase
After rearing with rapamycin, Day 14

Fig. S4. Chloroacetate esterase histochemistry of gill cells from the mussels reared with or without rapamycin. (**A**) Fluorescence micrographs (left) show DAPI (blue) stained symbionts in symbiosomes in gill sections from the mussels reared without rapamycin under methane-depleted conditions for 0, 7, 14, and 28 days. Bright-field micrographs show positive for chloroacetate esterase staining (blue) (middle) or double staining with butyrate (brownish-red) and chloroacetate esterase (right) in symbiosomes of bacteriocytes. High magnification of bright-field micrographs shows the esterase activities in symbiosomes harboring symbionts from the mussels reared for 7 days (inset). (**B**) Fluorescence micrographs (left) show DAPI (blue) stained symbionts in symbiosomes in gill sections from the mussels reared with rapamycin under methane-depleted conditions for 7, 14, and 28 days. Bright-field micrographs show negative for chloroacetate esterase staining (middle) of double staining with butyrate and chloroacetate esterase (right) in bacteriocytes of mussels reared with rapamycin. (**C**) Fluorescence micrographs (left) show DAPI (blue) stained the symbionts in symbiosomes in gill sections from the mussels reared without rapamycin for 14 days after rearing with rapamycin for 14 days. Bright-field micrographs show positive for chloroacetate esterase staining (blue) (middle) or double staining with butyrate (brownish-red) and chloroacetate esterase (right) in symbiosomes of bacteriocytes. Arrowheads indicate the bacteriocytes exhibiting butyrate esterase activity in symbiosomes harboring symbionts. It is noted that the double staining with DAPI was not performed because the chloroacetate esterase staining quenched the fluorescence signal in DAPI; Scale bars, 10 μm.

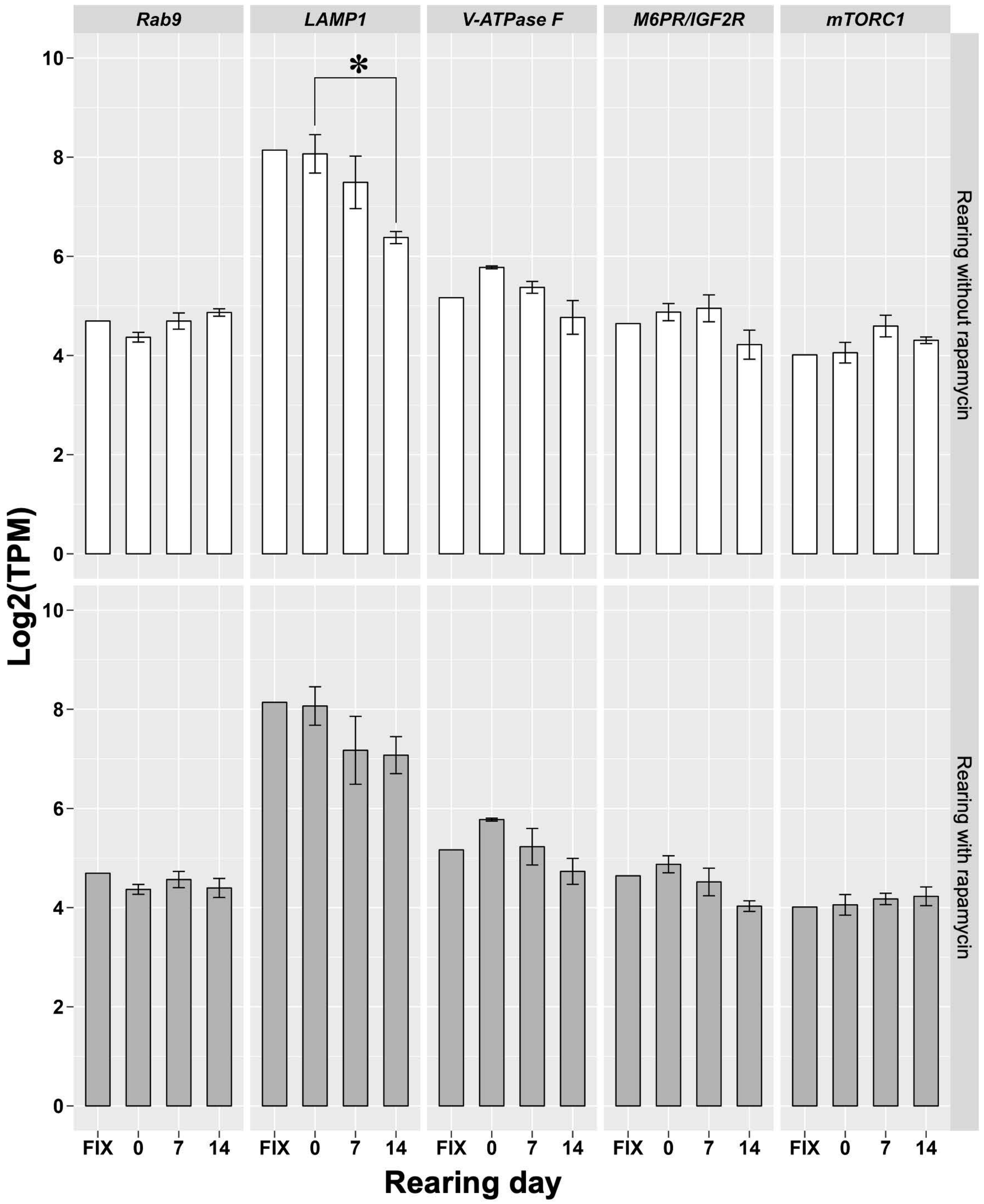

Fig. S5. Comparison of the target gene expressions using antibodies for immunohistochemistry. Expression patterns of genes of Rab9, LAMP1, V-ATPase, M6PR, and mTORC1 in the gills during the rearing with (lower) or without (upper) rapamycin under methane-depleted conditions for 0, 7, 14, and 28 days, including in situ fixed individual. Bars indicate mean ± standard deviation from three mussels. Values with an asterisk represent the significant differences (p.adjust value < 0.01) between rearing days. There were no significant differences between rearing with and without rapamycin. *in situ* fixed individual (FIX), rearing day 0, rearing day 7, as well as rearing day 14.

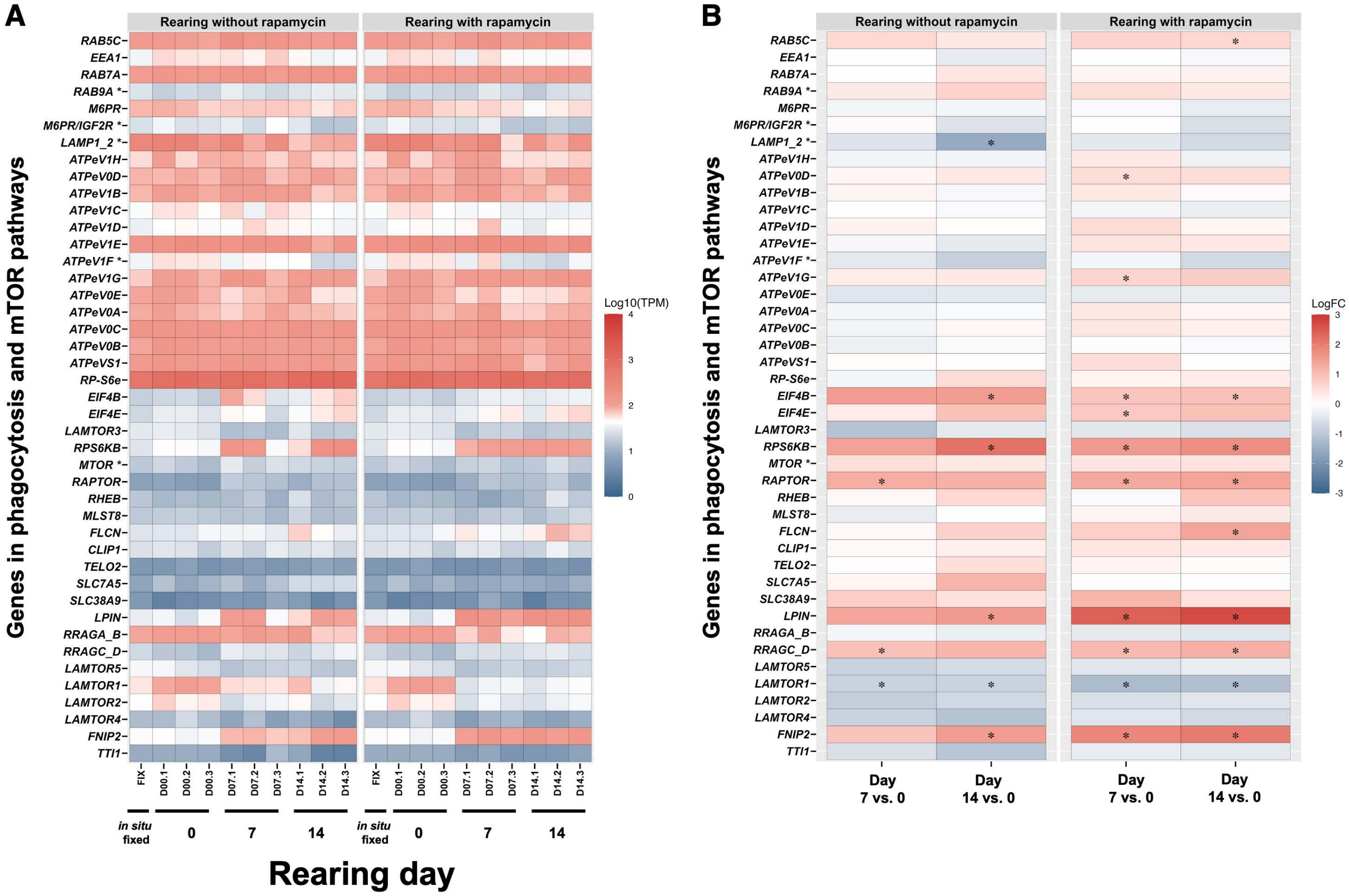

Fig. S6. Comparison of genes expression of mTOR signaling and phagocytosis during the rearing with or without rapamycin in gills of *Bathymodiolus japonicus*. (**A**) Expression patterns of the genes related to phagocytosis and mTORC1 pathways listed in Table S4 during rearing mussels, including in situ fixed individuals. The mussels were reared with or without rapamycin under methane-depleted conditions for 0, 7, and 14 days. For each rearing day, three individual mussels were examined. The color in the heatmap represents the values of Log10 (transcripts per million (TPM)) in genes. (**B**) Differentially expressed genes when comparing the rearing days. The heatmap colors represent the LogFC (log2 fold-change) value of day 0 vs. 7 and day 0 vs. 14. The genes marked with an asterisk were those with a p.adjust value below 0.01, significantly different between the rearing days in DEG analysis.

Table S1. Summary of dual transcriptome analysis in the host mussel *Bathymodiolus japonicus* and its methane-oxidizing symbionts.

Table S2. Genes for the biosynthesis of steroid and amino acid in the gills of *Bathymodiolus japonicus* and methane-oxidizing symbionts.

Table S3. Genes for compound transport in the gills of *Bathymodiolus japonicus* and methane-oxidizing symbionts.

Table S4. Genes for phagocytic and mTORC1 signaling pathway in *Bathymodiolus japonicus*.

Table S5. Comparison of the genes among bivalves genomes in phagocytosis and mTORC1 signaling pathway detected in *Bathymodiolus japonicus*.
